# Streamer Propagation along the Insulator with the Different Curved Profiles of the Shed

**DOI:** 10.3390/polym14050897

**Published:** 2022-02-24

**Authors:** Xiaobo Meng, Liming Wang, Hongwei Mei, Bin Cao, Xingming Bian

**Affiliations:** 1 School of Mechanical and Electrical Engineering, Guangzhou University, Guangzhou 510006, China; mengxb@gzhu.edu.cn; 2 Tsinghua Shenzhen International Graduate School, Tsinghua University, Shenzhen 518055, China; huhu0512@126.com; 3 The State Key Laboratory of Alternate Electrical Power System with Renewable Energy Sources, North China Electric Power University, Beijing 102206, China; bianxingming@ncepu.edu.cn

**Keywords:** streamer discharge, curved profiles, streamer propagation “stability” field, streamer propagation path, streamer propagation velocity

## Abstract

The flashover along the insulator endangers the reliable operation of the electrical power system. The reasonable curved profiles of the shed could improve the flashover voltage, which would reduce power system outages. The research on the influence of the curved profiles of the shed on the streamer propagation along the insulator made of polymer was presented in the paper. The streamer propagation “stability” field, path, and velocity affected by the curved profiles of the shed, were measured by ultraviolet camera, ICCD camera, and photomultipliers. The “surface” component of the streamer is stopped at the shed with the different curved profiles, while the “air” component could go round the shed and reach the cathode. The streamer propagation “stability” fields are inversely proportional to the curved profiles of the shed. The streamer propagation velocities are proportional to the curved profiles of the shed. The relationship between the streamer propagation and the flashover propagation was discussed in depth. The subsequent flashover propagation is greatly affected by the streamer propagation path and “stability” field. Furthermore, the influence of the material properties on the streamer propagation path was also discussed in depth.

## 1. Introduction

The flashover along the insulator surface causes many widespread power outages every year. It endangers the stability and safety of electrical power system, resulting in a large number of social and economic losses. The reasonable shed design might improve the flashover voltage, which would reduce power system outages. The test results of the flashover along the insulator with the different shed configurations have been used to optimize the design of the insulator by many researchers at home and abroad [1,2,3,4].

The flashover along the insulator was supported by many researchers [5,6,7]. However, the streamer discharge along the insulator with a shed were little investigated [8,9,10]. The streamer propagation along the insulator is the important physical process before the flashover. The inhibited streamer discharge along the insulator can lead to the subsequent flashover process to disappear. Hence, the research on the streamer discharge along the insulator with the different sheds is helpful for designing the shed configuration of insulator.

The streamer discharge along the smooth insulator have been investigated widely, but the characteristics of streamer discharge along the insulator with a shed were rarely researched [11,12,13,14,15]. In the paper [12,13], the influences of the shed on the streamer discharge were discussed, the “surface” component of streamer propagation along the insulator was blocked at the shed, but the “air” component could cross the shed and reach the cathode plate. In the paper [15], the influences of the shed configuration on the streamer discharge were discussed and summarized, such as the diameter, location, and combination of the shed. However, the influences of the curved profiles of the shed on the streamer discharge was not studied in depth. The photograph of streamer discharge along the profiled insulator surfaces was not shot by advanced high-definition camera [13]. The relation between the streamer discharge and flashover along the insulator with the shed was still not very clear [16].

In the paper, the streamer propagation “stability” field, path, and velocity affected by the curved profiles of the shed, were measured by ultraviolet camera, ICCD camera, and photomultipliers. The streamer energy loss at the shed was estimated by a method, which was used to interpret physical phenomenon in the test. Furthermore, the relationship between the characteristics of the streamer and the flashover was discussed in depth. It provided theoretical and experimental basis for optimization design of the curved profiles of the shed.

## 2. Experimental Arrangement and Measurement System

**Figure 1:**
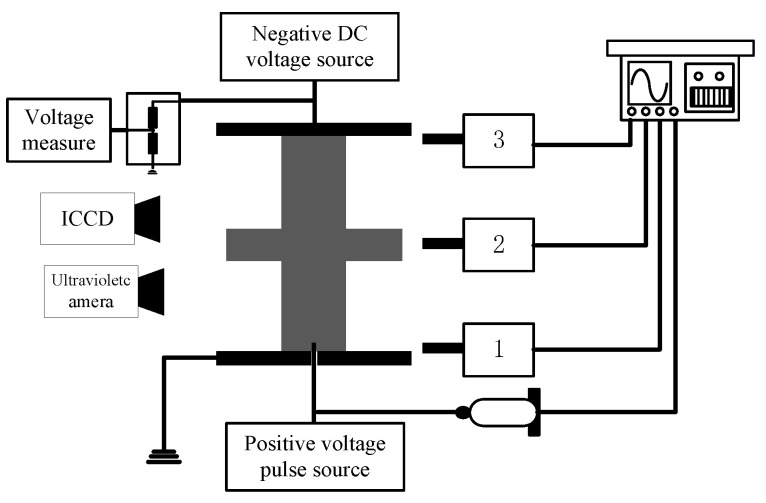
Experimental equipment.

The streamer propagation “stability” field, path, and velocity affected by the curved profiles of the shed, were measured by ultraviolet camera, ICCD camera, and photomultipliers in a three-electrode arrangement. The experiment arrangement and measurement system could be found in our past literature [15], as shown in Figure 1. The streamer discharge was triggered by a square pulse voltage applied to the needle electrode. A negative DC voltage was applied to two flat electrodes to produce a uniform electric field. The photographs of the streamer discharge paths were shot by the UV imaging detector (Ofil Corporation, Tel Aviv-Yafo, Israel). The micromorphology of streamer discharge was taken by an ICCD camera (Princeton Instruments, Trenton, NJ, USA). The photons radiated from the streamer discharge could be acquired by the photomultiplier (ET Enterprises Limited, Uxbridge, UK), so three photomultipliers were adopted to monitor the development process of the streamer discharge.

**Figure 2:**
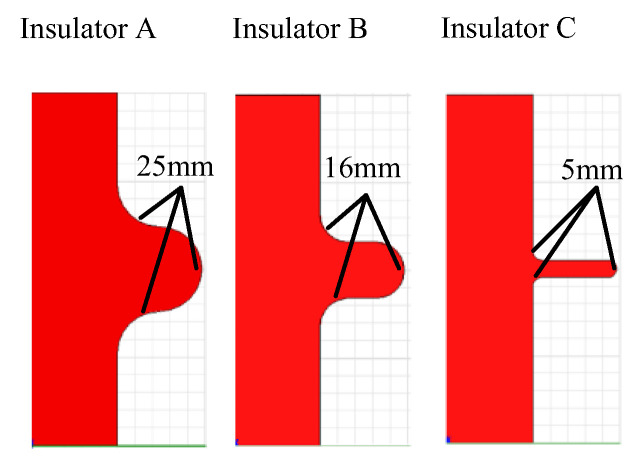
Insulators with the different fillet diameters of the curved profiles of the sheds.

The experiment was taken under standard atmospheric pressure, and the relative humidity was about 60% and temperature was about 20 °C. The insulators with the different curved profiles of the shed were made of polymer (nylon). The diameters of the sheds were 70 mm. The fillet diameters of the curved profiles of the sheds were 25 mm, 16 mm, and 5 mm respectively, as shown in Figure 2. The surface resistivity of the polymer (nylon) is 6.8 × 10^9^ Ω under the test condition.

## 3. Experimental Results

### 3.1. Streamer Propagation Fields

**Figure 3:**
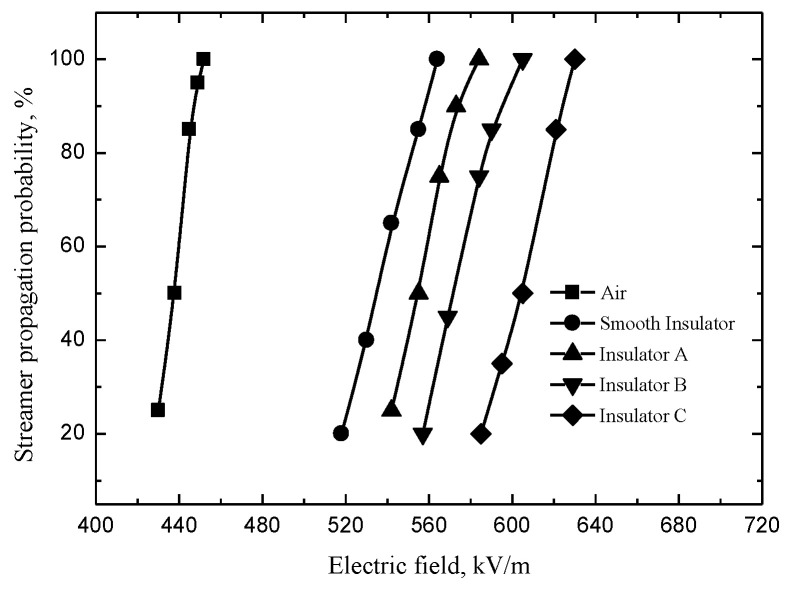
Streamer propagation probability.

The streamer propagation probability under the different electric fields is an important parameter in the development of streamer, which is useful to reveal streamer characteristics. The detailed measurement method of the streamer propagation probability was based on our past literature [17]. The streamer propagation probability distribution is shown in Figure 3.

**Figure 4:**
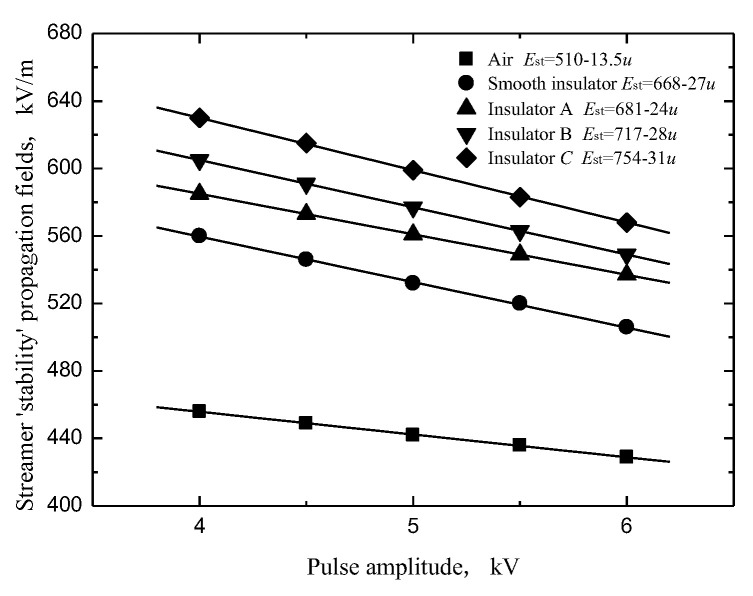
Stability fields for streamer propagation.

The formula of Gaussian distribution could be used to calculate the streamer “stability” propagation fields *E*_st_ corresponding to streamer propagation probability of 97.5% [15]. Figure 4 shows that the relation between the pulse amplitude and the streamer “stability” propagation fields is linear. There is inverse proportional relationship between the streamer “stability” propagation fields and the pulse amplitude, as same as the result in the literature [18]. The streamer “stability” propagation fields for the insulator with a shed are larger than that for the smooth insulation surface and the air alone. Specifically, the streamer “stability” propagation fields are inversely proportional to the fillet diameters of the curved profiles of the sheds.

### 3.2. Light Emission

The photomultipliers were used to measure the streamer discharge along the insulators with the different curved profiles of the sheds. The double light peaks were detected by the photomultiplier 2 at the shed, whereas single light peak at the cathode were detected by the photomultiplier 3. The test results measured by the photomultiplier have been discussed in depth in the literature [15]. The “surface” component of streamer propagation along the insulator is blocked at the shed, but the “air” component could cross the shed and reach the cathode plate.

**Figure 5:**
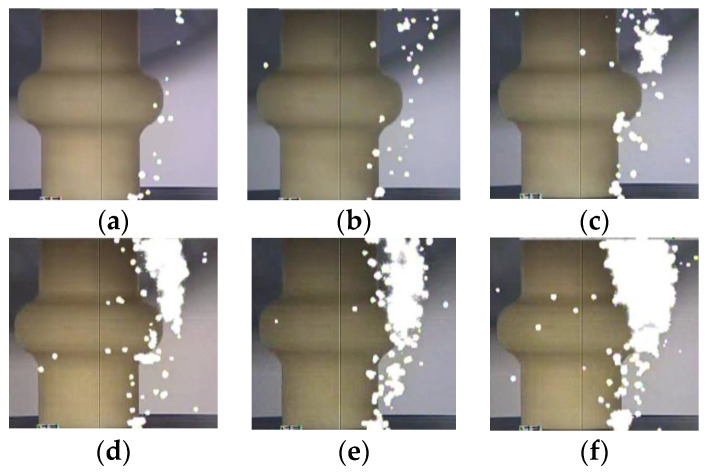
Streamer discharge photographs (Insulator A). (**a**) 570 kV/m, (**b**) 590 kV/m, (**c**) 630 kV/m, (**d**) 680 kV/m, (**e**) 710 kV/m, (**f**) 740 kV/m.

**Figure 6:**
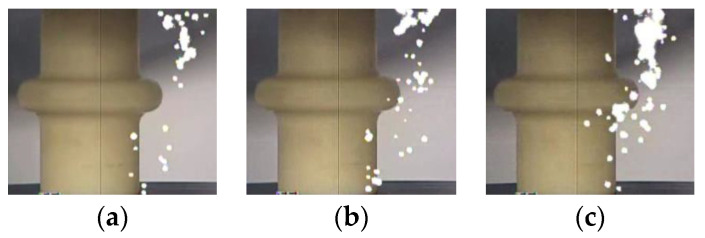

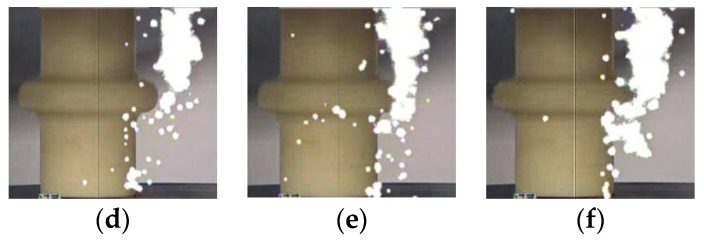
Streamer discharge photographs (Insulator B). (**a**) 610 kV/m, (**b**) 630 kV/m, (**c**) 650 kV/m, (**d**) 690 kV/m, (**e**) 710 kV/m, (**f**) 750 kV/m.

**Figure 7:**
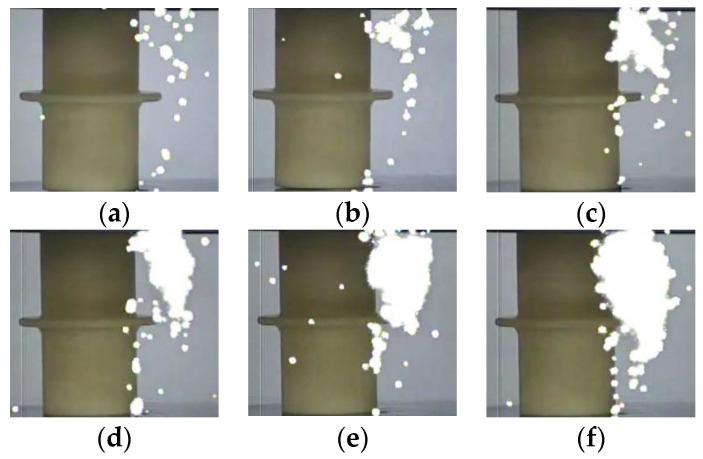
Streamer discharge photographs (Insulator C). (**a**) 630 kV/m, (**b**) 650 kV/m, (**c**) 690 kV/m, (**d**) 720 kV/m, (**e**) 750 kV/m, (**f**) 780 kV/m.

The photographs of the streamer discharge along the insulators were shot by the ultraviolet camera (Figure 5, Figure 6 and Figure 7). The shooting method of the ultraviolet camera was listed in the literature [17]. Consistent with the measurement result of the photomultipliers, the “air” propagation path bypasses the shed and reaches the cathode, while the “surface” propagation path stops at the shed.

**Figure 8:**
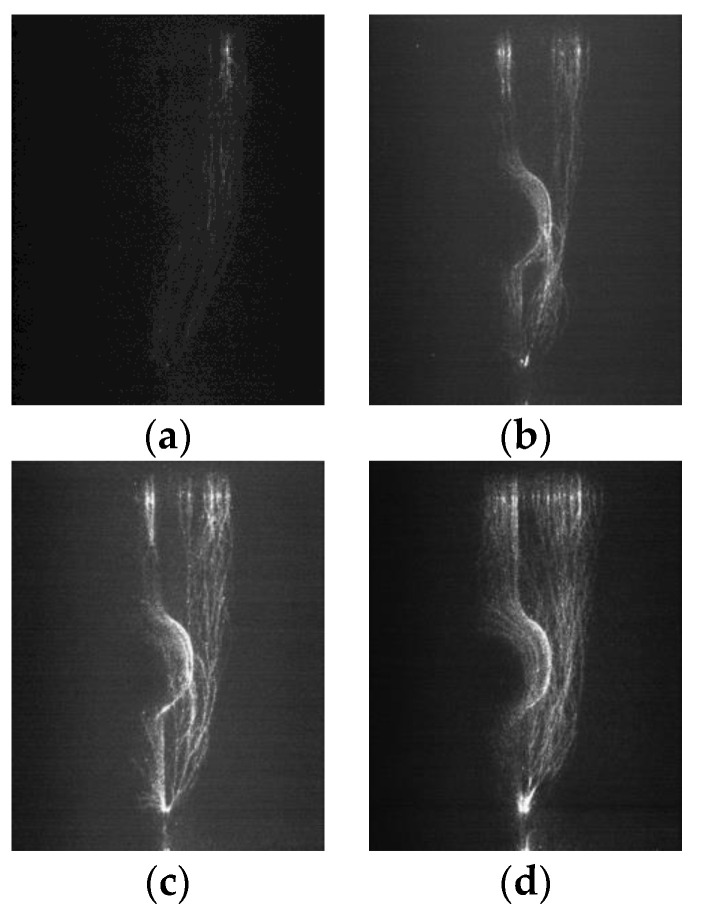
Streamer propagation photographs measured by ICCD camera (Insulator A). (**a**) 618 kV/m (**b**) 643 kV/m, (**c**) 690 kV/m, (**d**) 720 kV/m.

**Figure 9:**
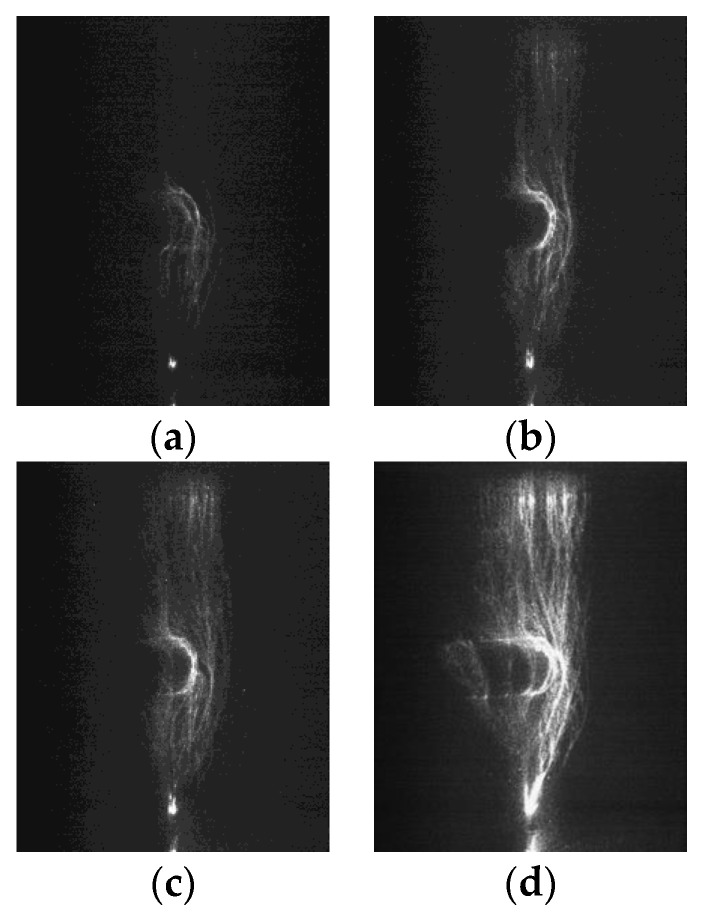
Streamer propagation photographs measured by ICCD camera (Insulator B). (**a**) 620 kV/m, (**b**) 660 kV/m, (**c**) 700 kV/m, (**d**) 740 kV/m.

**Figure 10:**
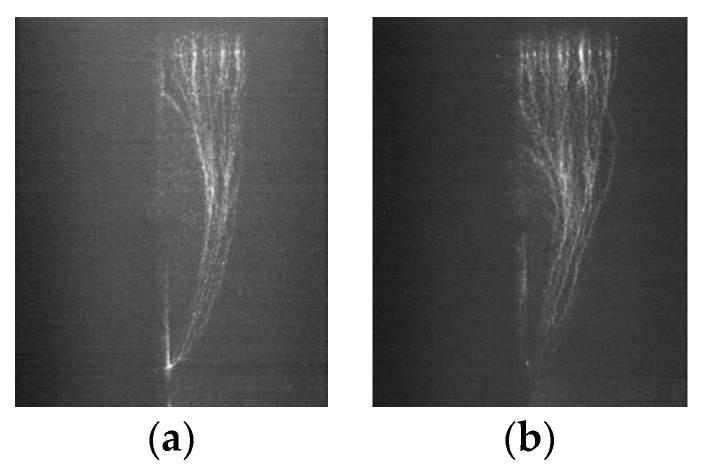

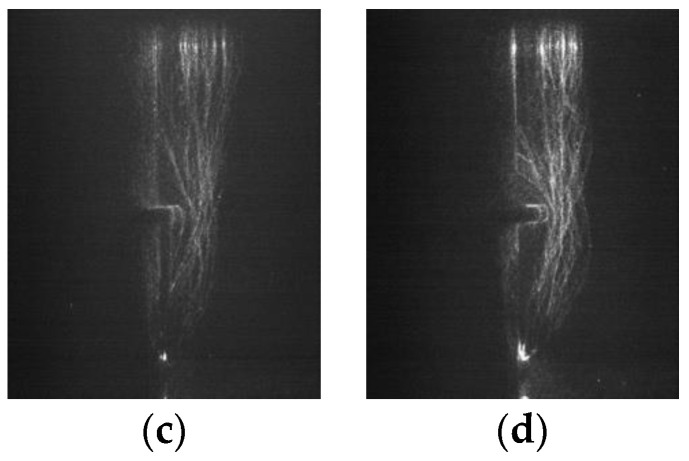
Streamer propagation photographs measured by ICCD camera (Insulator C). (**a**) 640 kV/m (**b**) 680 kV/m, (**c**) 720 kV/m (**d**) 760 kV/m.

**Figure 11:**
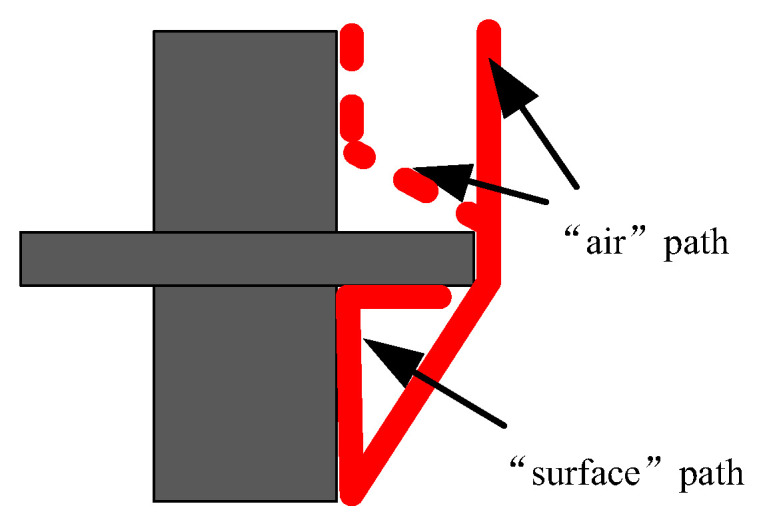
Streamer propagation paths along the insulators.

The ICCD camera (PI MAX3) made by Princeton Instruments was used to take photographs of the micromorphology of the streamer propagation along the insulators. Due to the weaknesses of the streamer discharge, each photograph recorded the three-times process of streamer discharges in order to make the photograph clearer. Figure 8, Figure 9 and Figure 10 shows the streamer discharge photographs. It is found that there are also two streamer paths: the “air” propagation path and the “surface” propagation path. The “air” propagation path crosses the shed and arrives at the cathode. Different from the measurement results of the ultraviolet camera, the “surface” propagation path also crosses the shed and arrives at the cathode. However, the photomultiplier 3 detected single light peak at the cathode, which meant that the “surface” component was blocked by the shed, and only the “air” component crossed the shed. The reason to explain this contradiction is that the “surface” component is blocked by the shed. When the “air” component of streamer bypasses the shed, sometimes it might propagate along the “surface” propagation path due to the attraction of the surface charge [19,20,21], or sometimes it propagated along the “air” propagation path, or both cases existed in the one streamer discharge. Due to the larger fillet diameters of the curved profiles of the insulators A and B, the air component is more easily attracted by the surface charge and develops along the insulating material surface. This situation is not obvious on the surface of the insulator C due to the smallest fillet diameters of the curved profiles. In the literature [15], the “air” component of streamer might propagate along either the “surface” propagation path or the “air” propagation path. Therefore, the “surface” propagation path and the “air” propagation path behind the shed both belong to the “air” component of streamer due to the attraction of the surface charge. The greater the fillet diameters of the curved profiles, the easier it is for the “air” component of streamer to develop along the surface of the insulating material. The streamer propagation paths along the insulator with a shed are described in Figure 11.

### 3.3. Streamer Propagation Velocity

**Figure 12:**
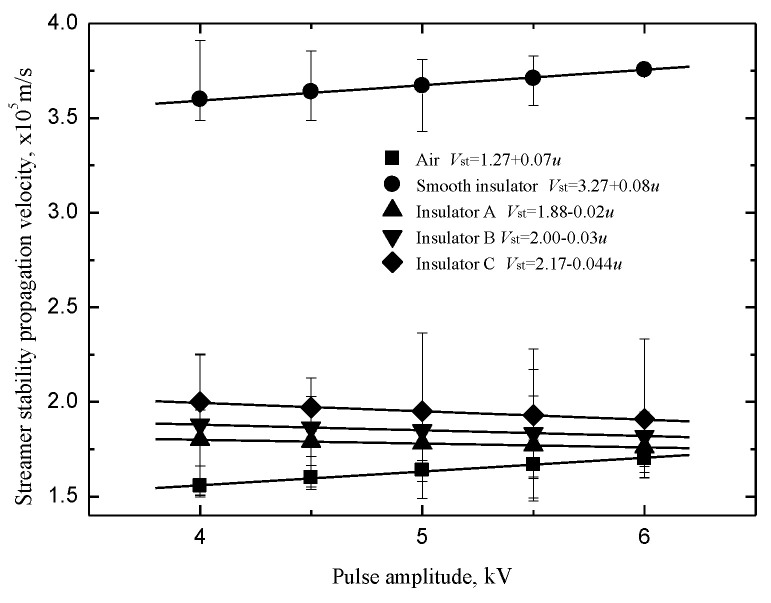
Streamer stability propagation velocity.

**Figure 13:**
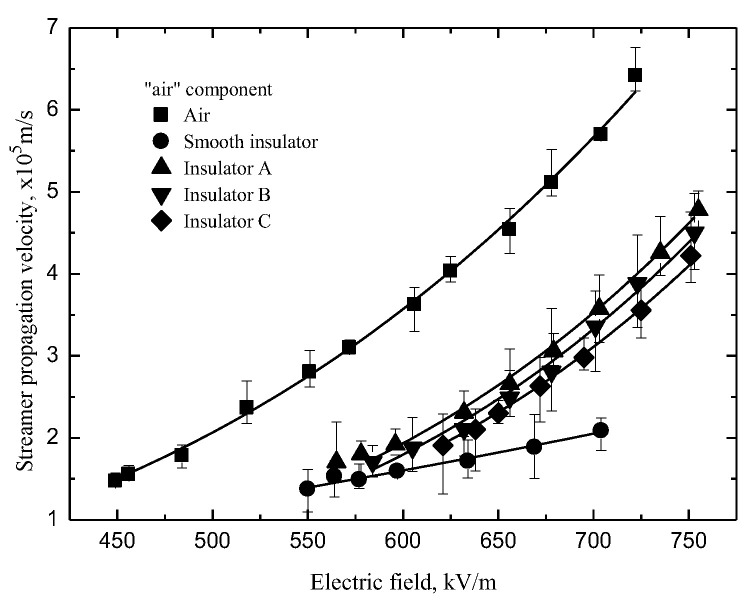
Streamer propagation velocity (“air” component).

The streamer “stability” propagation velocities *V*_st_ were defined as the velocities of streamer propagation at the stability fields [22]. The velocities of streamers propagating along the insulators with a shed decreases linearly but slowly with the pulse amplitude in Figure 12. The velocities of the “air” component along the insulators with a shed are higher than that along the smooth insulator in Figure 13. The reason could be found in the literature [15]. It is obvious that the “air” component velocities are proportional to the fillet diameters of the curved profiles of the sheds.

### 3.4. Evolution of Streamer to Flashover

The characteristics of flashover along the insulator were investigated through improving the applied voltage between the plates. The flashover “stability” propagation fields *E*_50_ corresponding to flashover propagation probability of 50% could be acquired by the method of acquiring the streamer “stability” propagation fields *E*_st_. Table 1 shows that the flashover “stability” propagation fields *E*_50_ increase with streamer “stability” propagation fields *E*_50_. The flashover “stability” propagation fields are also inversely proportional to the fillet diameters of the curved profiles of the sheds. Hence, the applied electric field has the same effect on the streamer propagation and the subsequent flashover propagation.

**Figure 14:**
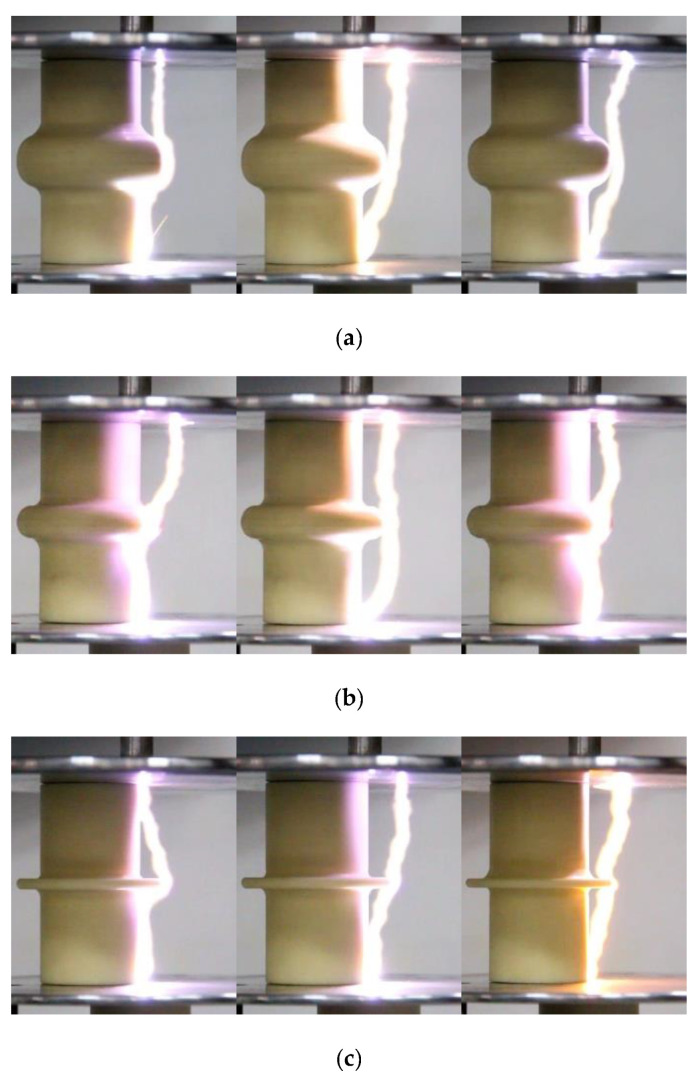
Photographs of flashover along the insulator with the shed. (**a**) Insulator A. (**b**) Insulator B. (**c**) Insulator C.

The photographs of the flashover path were acquired by a high-speed camera in Figure 14. It shows that the flashover propagates along the insulator with only one propagation path. In front of the shed, the flashover propagates along either the “surface” or “air” propagation path of the previous streamer. The flashover propagated along the “air” propagation path of the previous streamer behind the shed due to the interruption of the “surface” propagation path at the shed. Hence, the streamer channel has a great influence on the subsequent flashover. The reasonably optimized curved profiles of the sheds could improve the electric fields for the streamer propagation, thereby, the electric fields for the flashover propagation are also improved. The perspective of restraining the streamer discharge to prevent the flashover discharge would be a new idea to design the curved profiles of the shed.

## 4. Discussion

### 4.1. Tangential Electric Field along Streamer Propagation Path

**Figure 15:**
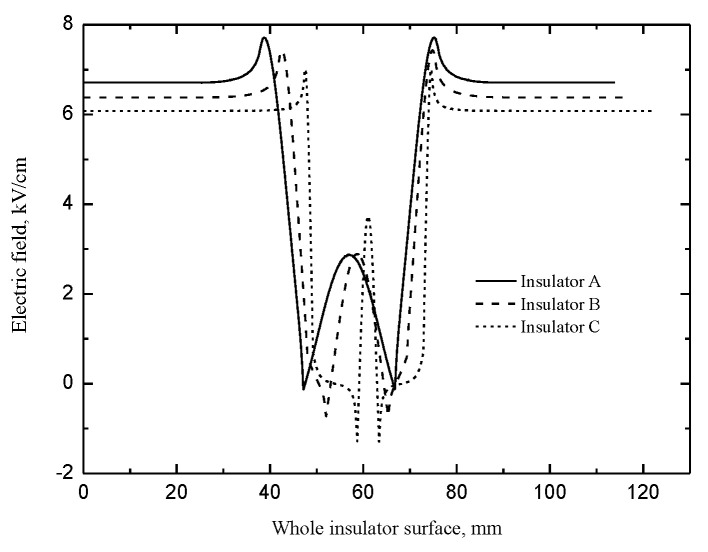
Tangential electric field along the whole insulator surface.

Ansoft Maxwell was adopted to calculate the electric fields along the insulator. Figure 15 shows the tangential electric field along the whole insulator surface. The tangential electric field is small and even negative in the regions of the curved profiles of the shed. The reason why the “surface” component of streamer would not cross this small electric field region is that the energy from this small electric field could not maintain the streamer propagation. Hence, the “surface” propagation path fails to reach the cathode.

**Figure 16:**
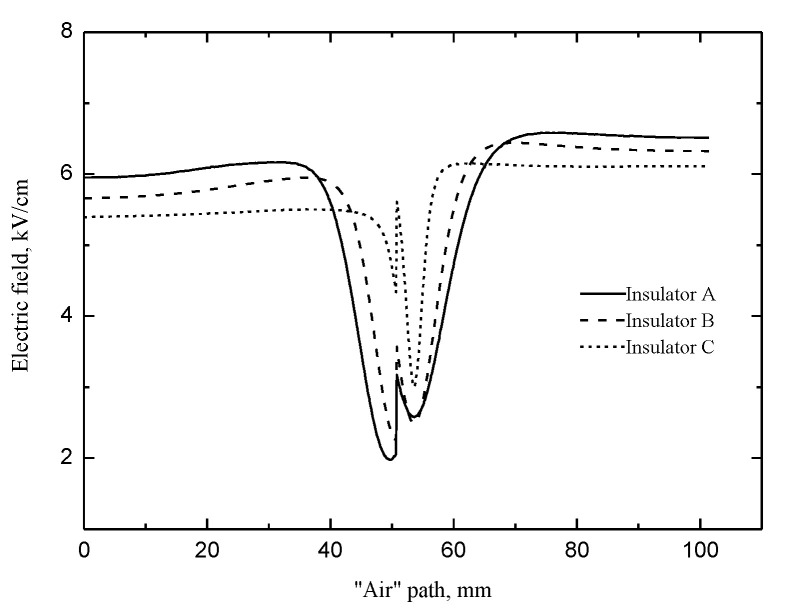
Tangential electric field along the “air” path.

However, the “air” component could cross the shed and reach the cathode plate. Hence, the streamer propagation characteristic parameters, such as the streamer “stability” propagation fields and the streamer “stability” propagation velocities, are all determined by the “air” component of streamer. The distribution of the tangential electric field along the “air” path is important to explain the streamer propagation characteristic parameters. Figure 16 shows that the tangential electric field along the “air” path before and behind the shed is proportional to the fillet diameters of the curved profile of the shed; whereas, the tangential electric field at the shed is inversely proportional to the fillet diameters.

The streamer has not yet obtained much energy from the electric field in front of the shed, so the applied electric field has the greatest impact on it. When the streamer propagates along the insulator with the largest fillet diameters of the curved profile of the shed, the tangential electric field along the “air” path is the largest, so it is easy for the streamer to develop and to cross the shed where the tangential electric field is greatly reduced. Therefore, lower electric field is required for the streamer to propagate along the insulator with the larger fillet diameters of the curved profile of the shed. Under the same electric field, the “air” component velocities along the insulator are proportional to the fillet diameters of the curved profiles of the sheds.

### 4.2. Streamer Propagation Energy Loss at the Shed

The streamer discharge would lose much energy at the shed due to the low tangential electric field and the charges in the streamer attaching to the shed [23,24,25,26]. The method in the literature [15] was used to estimate the energy loss at the shed. The calculated energy loss at the shed (*L*_shc_) differed by an error (*Q*_ste_) from the real energy loss (*L*_shc_) as shown in Equation (1).

(1) $$L_{\mathrm{sh}}=L_{\mathrm{shfc}}+L_{\mathrm{shsc}}-\left(Q_{\mathrm{stfe}}-Q_{\mathrm{stse}}\right)=L_{\mathrm{shc}}-Q_{\mathrm{ste}}$$

In Table 2, *E*_st1_ is the stability fields for streamer propagation along insulators with a shed. *v*_1_/*v*_2_ is the “air” component velocity before/after the shed at *E*_st1_. *Q*_Ef1_/*Q*_Es1_ is the energy obtained from ambient electric field before/after the shed. *E*_f2_ is the ambient field corresponding to the same value of *v*_1_ when streamer propagates along smooth insulators during the first half part of air gap. *E*_s2_ is the ambient field corresponding to the same value of *v*_2_ when streamer propagates in air alone during the second half part of air gap. *Q*_Ef2_/*Q*_Es2_ is the energy obtained from ambient electric field *E*_f2_/*E*_s2_ in its case.

Table 2 showed the energy loss at the shed (*L*_shc_) is inversely proportional to the fillet diameters of the curved profile of the shed. From the perspective of the energy loss, the streamer propagation energy loss at the shed is inversely proportional to the fillet diameters of the curved profile of the shed. Therefore, the streamer “stability” propagation fields are inversely proportional to the fillet diameters, and the streamer propagation velocities are proportional to the fillet diameters under the same electric field.

### 4.3. Influence of the Material Properties

In the literature [12,13], the authors found that the “air” component of streamer discharge could bypass the shed and reach the cathode, the “surface” component of streamer discharge could also bypass the shed with the large fillet diameters of the curved profile and reach the cathode. However, we used more advanced experimental equipment and experimental testing methods in this paper, such as the ICCD camera and the UV imaging detector was adopted to shoot the streamer discharge photographs. The different test conclusion was obtained that the “surface” component of streamer discharge could not bypass the shed, even the fillet diameters of the curved profile of the shed is much large. The “surface” propagation path behind the shed does not belong to the “surface” component of streamer discharge, but belongs to the “air” component of streamer discharge.

**Figure 17:**
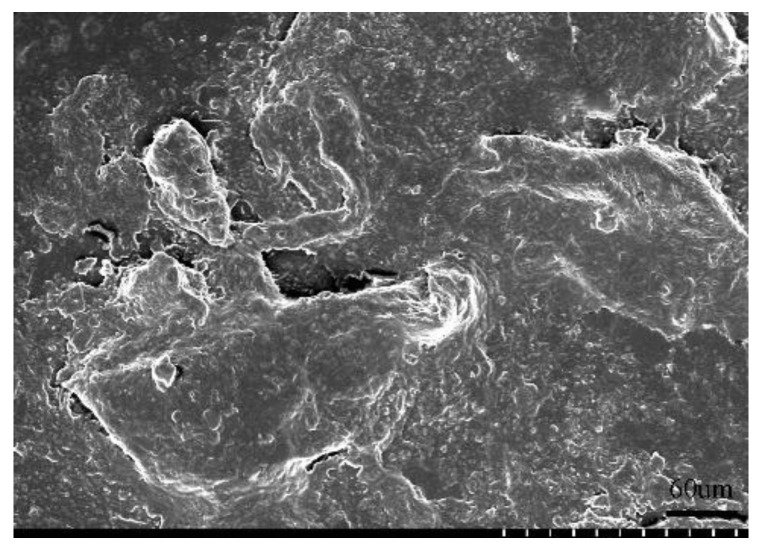
Scanning electron microcopy images.

Our conclusion is closer to the actual situation, in addition to our more advanced measuring equipment, it is more important that we consider the influence of material properties on the streamer discharge. There are much microporous defects (physical defect and chemical defect) on the surface of the polymer in Figure 17 measured by the scanning electron microscope. The surface adsorbed charges on the polymer increase with the increase of the microporous defects [27]. Therefore, the surface of the polymer would adsorb a lot of negative charge with the negative DC voltage applied on the gap.

**Figure 18:**
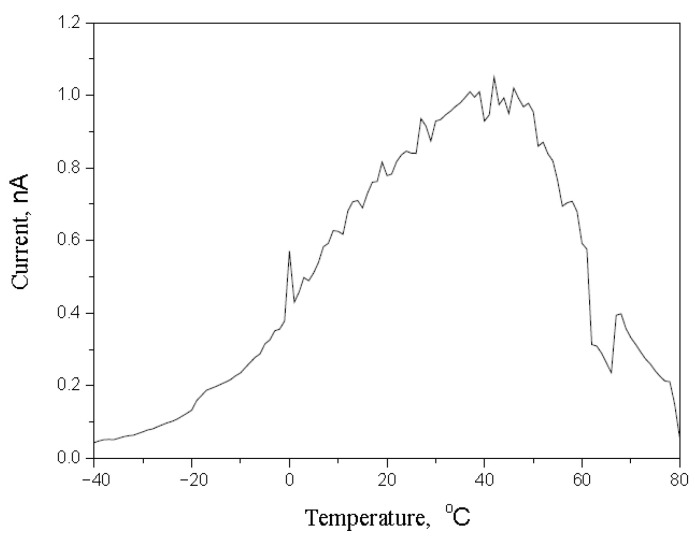
The result of the thermally stimulated current.

The method of the thermally stimulated current was used to measure the trap charges (nC) on the surface of the polymer. The trap charges on the polymer could be calculated by the result of the thermally stimulated current in Figure 18. The trap charge on the polymer used in the test is 1879 nC, which is greater than other materials. The more trap charges means that the more negative charge is accumulated or deposited on the polymer surface. The result of the thermally stimulated current proves the result of the scanning electron microscope.

**Figure 19:**
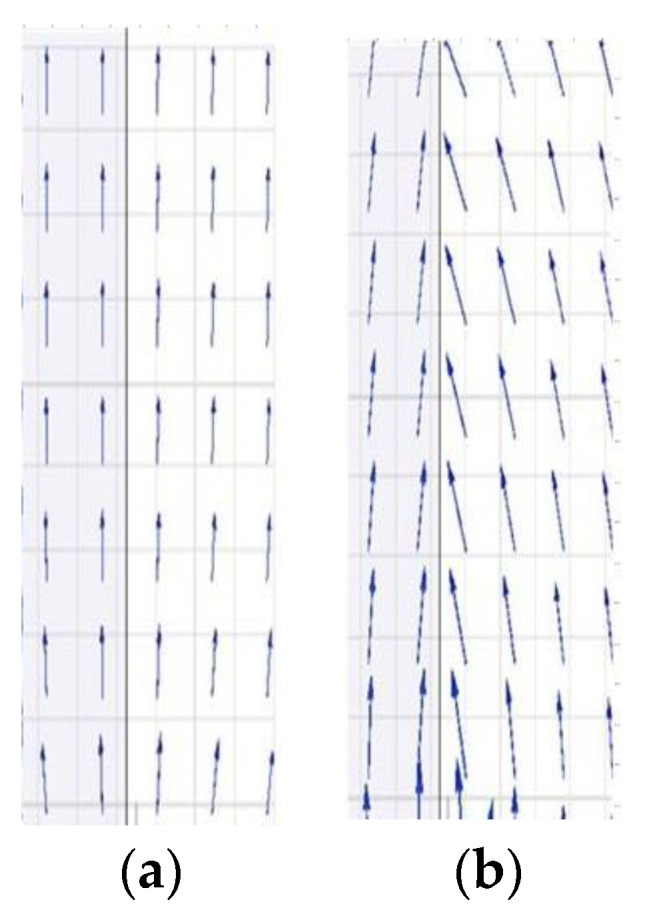
Vector plots of the electric field distribution along the surface of the polymer. (**a**) no surface charge, (**b**) −10 μC/m^2^ surface charge. The darker part is polymer material, the white part is air.

As shown in Figure 19, the vector of electric fields is parallel to the surface of the polymer with no surface charge. However, the vector of electric fields points to the surface of the polymer with negative surface charge. Therefore, the air component of streamer discharge behind the shed is more easily attracted by the polymer surface and develops along the “surface” propagation path. It could explain reasonably that why the “surface” propagation path and the “air” propagation path behind the shed both belong to the “air” component of streamer in the Section 3.2.

This contradiction of the measurement result from the ICCD camera and the UV imaging detector has given us great enlightenment. It is easy to produce confusing results or conclusions for a single test equipment or measurement method to study the physical process of the temporal and spatial evolution of streamer discharge. Combining a variety of test equipment or measurement methods, and then comprehensively analyzing the test results, can accurately reveal the physical development process of streamer discharge.

The test results tell us that the smaller fillet diameters of the curved profile of the shed would be adopted to obtain the larger flashover voltage in electrical power system. However, the outdoor insulators of the transmission lines are prone to accumulating pollution on the surface in the atmospheric environment, and the larger fillet diameters of the curved profile of the shed would remove pollutants easily through rain and wind. Hence, the fillet diameters of the curved profile of the shed not only affect the flashover voltage of the insulator, but also the removal of pollutants through rain and wind. In engineering practice, the fillet diameters of the curved profile of the shed should be chosen to consider the impact on both sides, taking into account both flashover voltage and cleaning capacity. Sometimes in order to obtain high cleaning ability, the flashover voltage can be appropriately reduced to obtain better environmental adaptability. These results provide a theoretical basis for promoting the shape of the shed.

## 5. Conclusions

From typical photomultiplier signals and the streamer discharge photographs, the “surface” component of streamer discharge was blocked at the shed. Only the “air” component crossed the shed, and the “air” component behind the shed might propagate along either the “surface” propagation path or the “air” propagation path.

The streamer “stability” propagation fields were inversely proportional to the fillet diameters of the curved profiles of the sheds. The streamer propagation velocities were proportional to the fillet diameters.

The applied electric field had the same effect on the streamer propagation and the subsequent flashover propagation. Furthermore, the streamer channel had a great influence on the development path of the subsequent flashover. The perspective of restraining the streamer discharge to prevent the flashover discharge would be a new idea to design the curved profiles of the shed.

The tangential electric field along the streamer propagation path and the streamer propagation energy loss at the shed could be used to explain the characteristics of the streamer discharge. From the perspective of the tangential electric field, when the streamer propagates along the insulator with the largest fillet diameters of the curved profile of the shed, the tangential electric field along the “air” propagation path is the largest, so it is easy for the streamer to develop and to cross the shed where the tangential electric field is greatly reduced. From the perspective of the streamer propagation energy loss, the streamer propagation energy loss at the shed is inversely proportional to the fillet diameters of the curved profile of the shed. Therefore, lower electric field is required for the streamer to propagate along the insulator with the larger fillet diameters of the curved profile of the shed.

The surface of the polymer would adsorb a lot of negative charge with the negative DC voltage applied on the gap. The vector of electric fields points to the surface of the polymer with negative surface charge. Hence, the air component of streamer discharge behind the shed is more easily attracted by the polymer surface and develops along the “surface” propagation path. It could explain reasonably that why the “surface” propagation path and the “air” propagation path behind the shed both belong to the “air” component of the streamer.

## Figures and Tables

**Table 1:** The comparison of streamer “stability” propagation fields Est and flashover “stability” propagation fields E50.

Shed Configuration	*E*_st_ (kV/m)	*E*_50_ (kV/m)
Insulator A	585	824
Insulator B	605	842
Insulator C	630	863

**Table 2:** Energy loss at the shed.

Shed Configuration	Insulator A	Insulator B	Insulator C
*E*_st1_(kV/m)	585	605	630
*v*_1_(10^5^ m/s)	1.4	1.45	1.52
*v*_2_(10^5^ m/s)	2.48	2.68	2.84
*Q*_Ef1_(10^4^ J/C)	2.93	3.03	3.15
*Q*_Es1_(10^4^ J/C)	2.93	3.03	3.15
*E*_f2_(kV/m)	580	595	610
*E*_s2_(kV/m)	527	535	550
*Q*_Ef2_(10^4^ J/C)	2.9	2.975	3.05
*Q*_Es2_(10^4^ J/C)	2.635	2.675	2.75
*L*_shc_(10^4^ J/C)	0.315	0.4	0.5

## Data Availability

Not applicable.
